# Recent advances and perspectives of postoperative neurological disorders in the elderly surgical patients

**DOI:** 10.1111/cns.13763

**Published:** 2021-12-03

**Authors:** Biying Liu, Dan Huang, Yunlu Guo, Xiaoqiong Sun, Caiyang Chen, Xiaozhu Zhai, Xia Jin, Hui Zhu, Peiying Li, Weifeng Yu

**Affiliations:** ^1^ Department of Anesthesiology State Key Laboratory of Oncogenes and Related Genes Shanghai Cancer Institute Renji Hospital School of Medicine Shanghai Jiao Tong University Shanghai China

**Keywords:** cognitive disorders, covert stroke, delirium, hemorrhagic stroke, postoperative neurological disorders, stroke

## Abstract

Postoperative neurological disorders, including postoperative delirium (POD), postoperative cognitive dysfunction (POCD), postoperative covert ischemic stroke, and hemorrhagic stroke, are challenging clinical problems in the emerging aged surgical population. These disorders can deteriorate functional outcomes and long‐term quality of life after surgery, resulting in a substantial social and financial burden to the family and society. Understanding predisposing and precipitating factors may promote individualized preventive treatment for each disorder, as several risk factors are modifiable. Besides prevention, timely identification and treatment of etiologies and symptoms can contribute to better recovery from postoperative neurological disorders and lower risk of long‐term cognitive impairment, disability, and even death. Herein, we summarize the diagnosis, risk factors, prevention, and treatment of these postoperative complications, with emphasis on recent advances and perspectives.

## Graphical abstract

This review described general features and the latest evidence‐based knowledge of postoperative neurological disorders with emphasis on recent advances and perspectives. This review mapped the existing evidence regarding risk factors, prevention, and treatment for postoperative neurological disorders to help guide future research and clinical management.

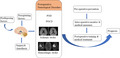

## 1 INTRODUCTION

Postoperative neurological disorders have been attracting increasing attention in the world, with a vast amount of research conducted on these poorly understood disorders. Postoperative neurological disorders contain postoperative delirium (POD), postoperative cognitive dysfunction (POCD), postoperative covert ischemic stroke, and hemorrhagic stroke, causing cognition decline and poor long‐term functional outcome in the elderly. Postoperative neurological disorders increase mortality and cause substantial financial burden on family and society. With a rapid increase in the number of elderly patients undergoing elective surgical procedures, postoperative neurological disorders need more concern and further investigation. This review aims to describe general features and the latest evidence‐based knowledge of postoperative neurological disorders.

## 2 POSTOPERATIVE NEUROLOGICAL DISORDERS

Postoperative neurological disorders include neurological complications such as delirium, cognitive dysfunction, acute cerebral ischemic stroke, and hemorrhagic stroke that occur after surgery, especially in the elderly. As more elderly patients undergo surgery, the incidence of postoperative neurological disorders is rapidly increased. Although their exact etiology and pathogenesis remain elusive, several risk factors have been recognized.

### 2.1 POD

Postoperative delirium is defined as acute emergence of confusion, disorientation, perceptual disturbances, emotional dysregulation, or sleep disturbances, manifesting within a certain period of time. The prevalence of delirium ranged from 5% to 50% when assessed with Confusion Assessment Method for the Intensive Care Unit (CAM‐ICU).^1^ POD can occur soon after general anesthesia and operation, for example, in the post‐anesthesia care unit (PACU). In many cases, POD is frequently linked to anesthesia.^2^ Compared to dementia which chronically deteriorates brain function, POD is usually acute, transient, and presenting common causative factors.^3^ It contributes to prolonged hospitalization, increased mortality rate, and reduced long‐term quality of life, which adds an additional burden to patients and families.

Besides DSM‐5 listed in Table 1 and ICD‐10 diagnostic criteria which supplements a disturbance in sleep‐awake cycle including insomnia, reverse of sleep‐awake cycle, various assessment tools have been developed to recognize POD.^4^ The CAM‐ICU is a screening tool which consists of the assessment of four characteristics: 1) acute onset and fluctuating course of mental state, 2) inattention, 3) disorganized thinking, and 4) altered level of consciousness.^5^ Delirium is diagnosed when both characteristics 1) and 2) are satisfied with 3) or 4) electively satisfied.^6^

**TABLE 1:** DSM‐5 Diagnostic criteria for delirium

DSM−5 Diagnostic criteria for delirium
A disturbance in attention (reduced ability to direct, focus, sustain, and shift attention) and awareness (reduced orientation to the environment). A disturbance that develops over a short period (usually hours to a few days) represents a change from baseline attention and awareness and fluctuates in severity during the day. An additional disturbance in cognition (memory deficit, disorientation, language. visuospatial ability, or perception). The disturbances in Criteria 1 and 3 are not better explained by another pre‐existing established or evolving neurocognitive disorder and do not occur in a severely reduced level of arousal, such as coma. There is evidence from the history, physical examination, or laboratory findings that the disturbance is a direct physiological consequence of another medical condition, substance intoxication or withdrawal (due to a drug of abuse or to a medication), or exposure to a toxin, or is due to multiple etiologies.

Abbreviation: DSM‐5, the Diagnostic and Statistical Manual of Mental Disorders, Fifth Edition

### 2.2 POCD

Postoperative cognitive dysfunction, also defined as postoperative neurocognitive disorder (pNCD),^7^, ^8^ is characterized by cognitive decline persisting for more than 30 days but less than 12 months following surgery. Unlike POD (Table 2), consciousness, orientation, and attention are not obviously affected in POCD.^9^ However, patients can still manifest impairment in memory, perceptual function, and language.^9^, ^10^ The incidence increases among the elderly, especially those over 60 years old.^11^ For elderly patients, cognitive decline may result in prolonged hospitalization, reduced quality of life, even increased mortality, which has been neglected in the assessment of patient's prognosis, especially for those undergoing general anesthesia and surgery.^12^ The diagnosis criteria for POCD are more complex than for POD, as POCD requires a subjective impression of postoperative cognitive decline in neuropsychological test.^13^

**TABLE 2:** Differential diagnosis of POD and POCD

	POD	POCD
Epidemiology	In all ages but more common in older people over 60	In all ages but more common in older people over 60
Manifestation	Disturbance in attention and awareness, emotion, cognition, and fluctuating severity of consciousness.	Cognitive deficits (impairment of memory, perceptual function, language, ability to combine tasks)
Diagnostic tools	Various delirium scale, for example, CAM‐ICU	Pre‐ and postoperative psychometric testing
Timing	Days to weeks	Persisting for months
Prognosis	Reversible if underlying causative factors are treatable	Reversible but with long‐time impairment

Abbreviations: POCD, postoperative cognitive dysfunction; POD, postoperative delirium.

### 2.3 Postoperative covert stroke

Cerebrovascular disease, a leading global cause of death and disability with approximately 6.2 million deaths due to stoke, is estimated to become the second leading cause of death by 2030.^14^ According to a systemic analysis for the Global Burden of Disease Study, the mortality rates caused by stroke range from 30.6% to 48.3%^15^ and are significantly related to operations.^16^ Covert stroke has been increasingly recognized over the years. It represents brain infarcts with silent and subtle manifestations that can be detected on brain imaging.^17^, ^18^ Covert stroke may contribute more to poor outcomes and prognosis in elderly patients presenting cognitive decline, as its subtle manifestation can lead to ignorance of cognitive symptoms.^19^

The incidence of postoperative covert stroke has gradually increased due to the aging population. So far, only a few studies have examined its mechanisms.^20^ Little is known about perioperative covert stroke except its association with substantially increased mortality.^21^ One multicenter prospective cohort study reported that postoperative covert stroke was found in 7% among 1114 participants over 65 years old who underwent inpatient, elective, noncardiac surgery, which were assessed with brain magnetic resonance imaging (MRI) after surgery and Montreal Cognitive Assessment (MoCA) on preoperative baseline and 1‐year follow‐up.^22^ Among the patients with a complete 1‐year follow‐up, cognitive decline after surgery occurred in 42% of participants who had postoperative covert stroke and 29% of participants who did not have postoperative covert stroke.^22^ In addition, another study suggested that covert stroke can increase the risk of POD, overt stroke, or transient ischemic attack (TIA) during one‐year follow‐up.^22^

### 2.4 Hemorrhagic stroke

Although hemorrhagic stroke comprises only 20% of all strokes, the perioperative hemorrhagic stroke could detrimentally deteriorate patients’ recovery and prognosis.^23^ According to the American Heart Association (AHA) and the American Stroke Association (ASA),^24^, ^25^ hemorrhagic stroke is divided into the following conditions: 1) focal bleeding in brain parenchyma and 2) in subarachnoid space or ventricular space due to rupture of blood vessel other than trauma. Perioperative hemorrhagic stroke may occur after cerebral hyper‐perfusion due to a sudden surge of blood pressure (BP) for a certain amount of time.^26^ Hypertension is the most common risk factor for hemorrhagic stroke.^27^ Most anesthetics induce hypotension, so they are unlikely to provoke hemorrhagic stroke while under anesthesia.^28^ However, if postoperative hypertension persists for several hours, it can lead to certain condition linked to a sudden surge of cerebral perfusion.^29^

What discussed above is a brief introduction of four major types of postoperative neurological disorders with current understanding, which present disorientation, memory deficit, changes in awareness and attention from the baseline. Cognitive decline and poor prognosis of elderly patients after surgery have been an increasing concern around the world. With few studies on postoperative neurological disorders, the mechanisms and pathophysiology remain unknown,^30^, ^31^ especially for POD, POCD, and covert stroke. Besides, various manifestations of postoperative neurological disorders add more difficulties for research. As postoperative complications become increasing concerns, the mechanisms, prevention, and management are focus points that still need further research.

## 3 RISK FACTORS OF ACUTE POSTOPERATIVE NEUROLOGICAL DISORDERS

The exact mechanisms and pathophysiology of POD, POCD, and postoperative stroke are unclear. In the following paragraphs, we discuss potential risk factors of postoperative neurological disorders.

### 3.1 Risk factors of POD and POCD

It is commonly accepted that interactions between predisposing factors and precipitating factors play an important role in the occurrence of postoperative neurological disorders.^32^ The smaller vulnerability a patient has, the less occurrence of neurological disorders.^32^ For example, as advanced age is a predisposing factor, patients over 65 years old may present POD or POCD when exposed to only a few precipitating factors.^33^ On the contrary, younger patients exposed to the same precipitating factors may not experience POD or POCD. The recent evidence‐based risk factors including some novel candidates for POD as listed in Table 3.^32^ POD and POCD share almost the same risk factors based on current limited researches.^34^ Moreover, SARS‐CoV‐2 (COVID‐19) infection, a new uprising disease, has been found to be a potential novel risk factor of POD and POCD during the pandemic in the past two years, which is related to an accelerated onset with neurological manifestations^35^ and deterioration of cognitive decline.^36^

**TABLE 3:** Risk factors of POD and POCD in perioperative patients

	Risk factors	Reference
Preoperative factors	Advanced age	32
	Comorbidities (eg, cerebrovascular including stroke, cardiovascular, peripheral vascular diseases, diabetes, anemia, Parkinson's disease, depression, chronic pain, anxiety disorders, renal failure, and alcohol use disorders)	126, 127, 128, 129, 130, 131, 132, 133
	Preoperative fluid fasting and dehydration	32, 131
	Preoperative blood transfusion	130
	Hyponatremia and hypernatremia	128
	Drugs with anticholinergic effects	32
	Quinolone	130
	Urine albumin to creatinine ratio (UACR)	134
Intraoperative factors	Site of surgery (abdominal and cardiothoracic)	126, 135
	Hybrid procedure	130
	Intraoperative bleeding	136
	Duration of surgery	126
	Bispectral index (too low or too high)	127
	Intraoperative electrolyte disturbance	128
	Hyperthermia or hypothermia	137, 138
	The rate of decline in intraoperative hemoglobin concentration	136
Postoperative factors	Pain	139
	Use of bed sensors	140
	Nursing home residency	141
	Delayed ambulation	129

Abbreviations: POD, postoperative delirium; POCD, postoperative cognitive dysfunction

### 3.2 Risk factors of postoperative stroke

Postoperative stroke remains one of the most serious complications after surgery. As for both ischemic stroke and hemorrhagic stroke, risk factors include conventional vascular risk factors, the type of surgery, and other perioperative events (Table 4). Vascular factors and nutritional state are important preoperative risk factors for stroke and postoperative stroke. Postoperative stroke happens more often in cardiovascular, general thoracic, and neurosurgery.^37^, ^38^ Among factors below, the BP and coagulation state are specific modifiable risk factors for postoperative stroke according to large‐scale database studies, which will be further discussed.

**TABLE 4:** Risk factors of perioperative stroke

	Risk factors	Reference
Preoperative factors	Vascular factors (eg, age, sex, history of stoke or TIA, arrhythmia, coagulopathy)	37, 41, 42, 142, 143, 144
	Anemia	42
	Malnutrition	
	Preoperative central nervous system malperfusion	145
	Cerebral diffusion‐weighted imaging lesions	146
	Renal dysfunction	147, 148
Intraoperative factors	Type of surgery (cardiovascular, neurosurgery, left pneumonectomy other types of surgery)	37, 38, 42
	Specific intraoperative events (arrhythmia, hypertension, hypotension)	44
Postoperative factors	Adverse events (cardiac arrest, severe arrhythmia)	149
	New‐onset atrial fibrillation	150, 151

Abbreviation: TIA, transient ischemic attack

#### 3.2.1 BP and stroke

Blood pressure fluctuation is an important risk factor for postoperative stroke.^39^ Emergency surgeries raise the incidence of neurological disorders and even affect long‐term cognitive functions.^40^ As mentioned before, older patients are usually complicated with underlying diseases including hypertension, coronary artery disease, and arrhythmia, and thus, they have a greater risk of suffering from postoperative neurological disorders.^37^, ^41^, ^42^ It has been reported that patients developing postoperative stroke (ischemic or hemorrhagic) have higher average mean arterial blood pressure (MAP >80 mmHg).^37^ Moreover, a previous study suggests a strong relationship between elevated pulse pressure and stroke, which increases cerebral vulnerability to ischemic stroke.^42^ Additionally, evidence showed that intraoperative hypotension (IOH) is associated with the risk of major postoperative cardiac or cerebrovascular events.^43^ The short exposure to MAP of 55–65 mmHg is significantly associated with postoperative adverse cerebrovascular events, while maintaining systolic blood pressure (SBP) within 10% of the reference value may prevent postoperative adverse events compared with standard care (only treating if SBP <80 mmHg or <40% of the reference value).^44^, ^45^

#### 3.2.2 Coagulation and stroke

For elderly patients, oral anticoagulants, including vitamin K antagonist (VKA) and non‐vitamin K oral antagonists (NOACs),^46^ are common and effective therapies for the prevention of thromboembolism and stroke. However, they may lead to an inherent risk of bleeding. For elective and urgent surgery, reversal of anticoagulants is a necessary process for perioperative management.^47^ Traditional broader anticoagulants VKAs, such as warfarin, while effective, had multiple dietary and drug interactions and a great risk for intracranial hemorrhage, where 4‐factor (II, VII, IX, and X) prothrombin complex concentrate (4F‐PCC) should be used for VKAs reversal.^48^ Recently new types of anticoagulants targeted specific clotting factors (factors IIa and Xa), including dabigatran and rivaroxaban, have been approved for anticoagulation use with less adverse effect and less risk of hemorrhage.^49^ In addition, it is recommended to monitor the coagulation state of patients during the perioperative period.^50^

Postoperative neurological disorders occur by the interaction of predisposing factors and precipitating factors. Risk factors of POD, POCD, and perioperative stroke are listed above, and there may be other underlying risk factors unknown. Therefore, more researches are needed to understand possible risk factors and pathophysiology related to postoperative neurological disorders. The preventive strategies and protocols need to be established for known risk factors as well.

## 4 PREVENTION

### 4.1 Perioperative prevention of POD

**FIGURE 1:**
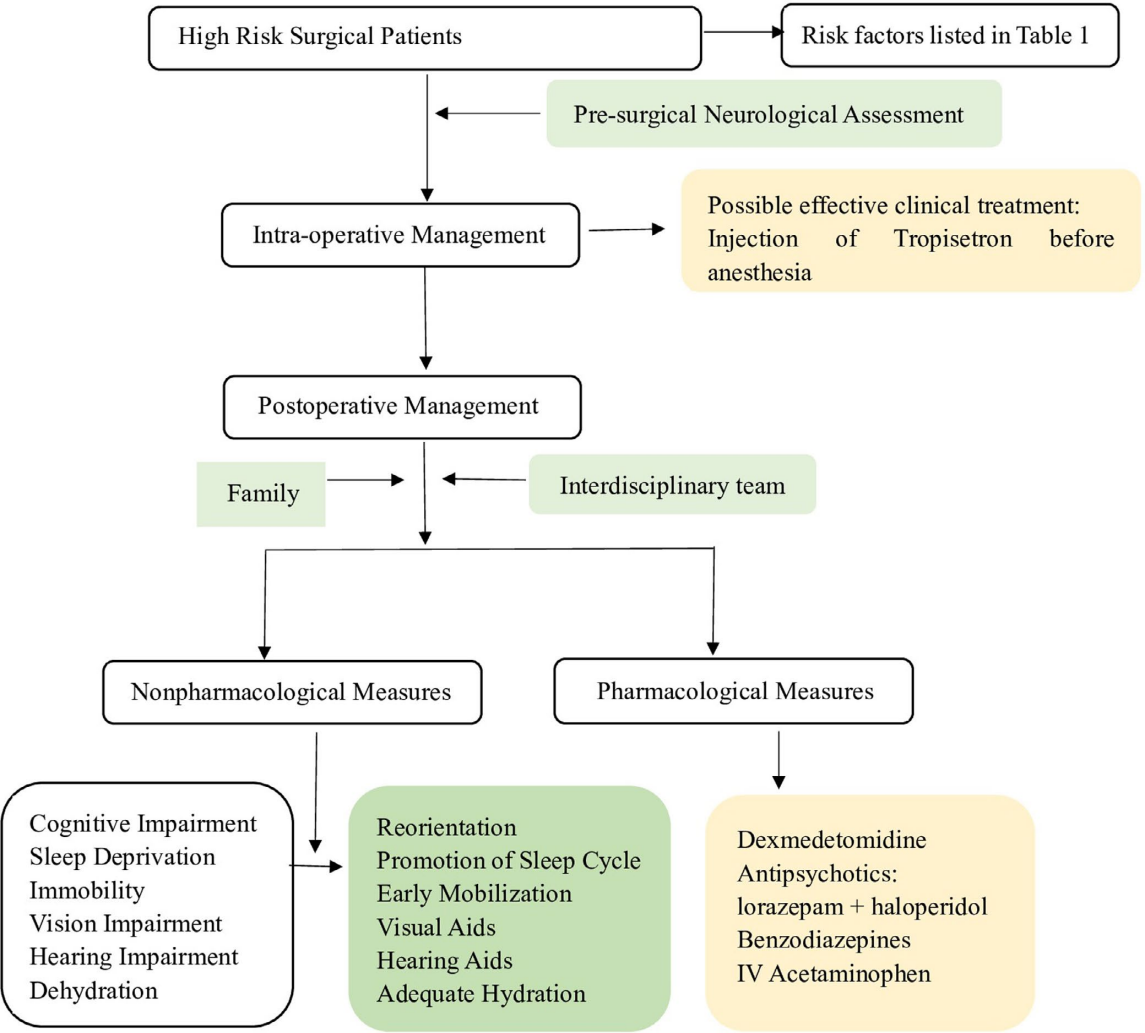
Nonpharmacological and pharmacological interventions during preventive process of POD recommended in HELP and latest evidence‐based advance research on prevention of POD, including interventions throughout the whole process of surgery and possible medications for treatment that needed further research. POD, postoperative delirium; HELP, Hospital Elder Life Program

Prevention strategies should be designed based on predisposing factors and parts of precipitating factors, which are the most effective measures against delirium.^51^ The Hospital Elder Life Program (HELP) released a multicomponent intervention guideline to prevent delirium^52^ (Figure 1).

#### 4.1.1 Nonpharmacological interventions

Based on the studies of HELP, the multicomponent nonpharmacological intervention significantly reduces the incidence of delirium, including reorientation (using orientation calendar, clocks), early mobilization, promotion of sleep cycle, adequate hydration, visual and hearing aids, and increased supervision in hospital.^53^ If implemented by a skilled interdisciplinary team, these measures are effective against POD.^54^ Apart from common contents of HELP, a recent clinical trial has found that tailored, family‐involved HELP could be beneficial for reducing POD, maintaining, or improving cognitive function, which may increase the implementation of this program.^55^ During the operation, however, the EEG (electroencephalography)‐guided anesthetic administration, compared with usual care, failed to decrease the incidence of POD.^56^

#### 4.1.2 Pharmacological interventions

Although several clinical trials have run various pharmacologic measures, there is a lack of strong evidence for effective prevention. One network meta‐analysis demonstrated that haloperidol plus lorazepam might be the best treatment, while ramelteon may be the best preventive medicine for POD.^57^ Besides, a recent randomized clinical trial has evaluated the effectiveness of tropisetron and found that tropisetron could decrease the incidence of delirium after noncardiac procedures in adults.^58^ Another trial has found that postoperative scheduled intravenous (IV) acetaminophen, combined with IV propofol or dexmedetomidine, could reduce the incidence of POD in hospital versus placebo group, while patients receiving one single intravenous anesthetic had no significant improvement for POD.^59^ According to a Cochrane review that examined antipsychotic medications for preventing delirium in hospitalized, non‐ICU patients, medications such as cholinesterase inhibitors, melatonin, and melatonin‐receptor agonists have no clear effect in preventing delirium.^60^

Besides, measures against precipitating factors are mainly adopted in the perioperative period, including limiting fasting time, reducing the use of preoperative medications such as benzodiazepines, opioids, and anticholinergic medications. For elderly patients with risk factors, preoperative neurological assessment is essential for predicting and preventing of POD.^61^

### 4.2 Prevention of POCD

#### 4.2.1 Preoperative interventions

The evaluation of patients’ baseline is important for the identification and prevention of POCD. Neuropsychological tests should be used before and after operations.^62^ Also, cognitive training and exercise^63^ have been proven to be beneficial for preventing POCD occurrence.^64^ Besides, Lu et al. found that pretreatment of parecoxib sodium combined with dexmedetomidine can decrease the incidence of POCD in patients undergoing arthroscopy by over 10%.^65^ These interventions should be particularly considered in high‐risk patients.

#### 4.2.2 Intraoperative interventions

In addition to preoperative interventions, there are several intraoperative measures to consider, including minimal exposure to anesthetics with careful monitoring. Regarding the anesthetic choice, Chen et al. found that the use of inhaled anesthetics^66^ in cardiac surgery generated higher postoperative scores in the Mini Mental State Exam (MMSE) compared with total intravenous anesthetics.^67^ Also, propofol may have a significant advantage in reducing POCD incidence compared with dexmedetomidine and midazolam sedation in elderly patients, in which midazolam has the highest inhibitory effects on cognitive functions.^68^

**FIGURE 2:**
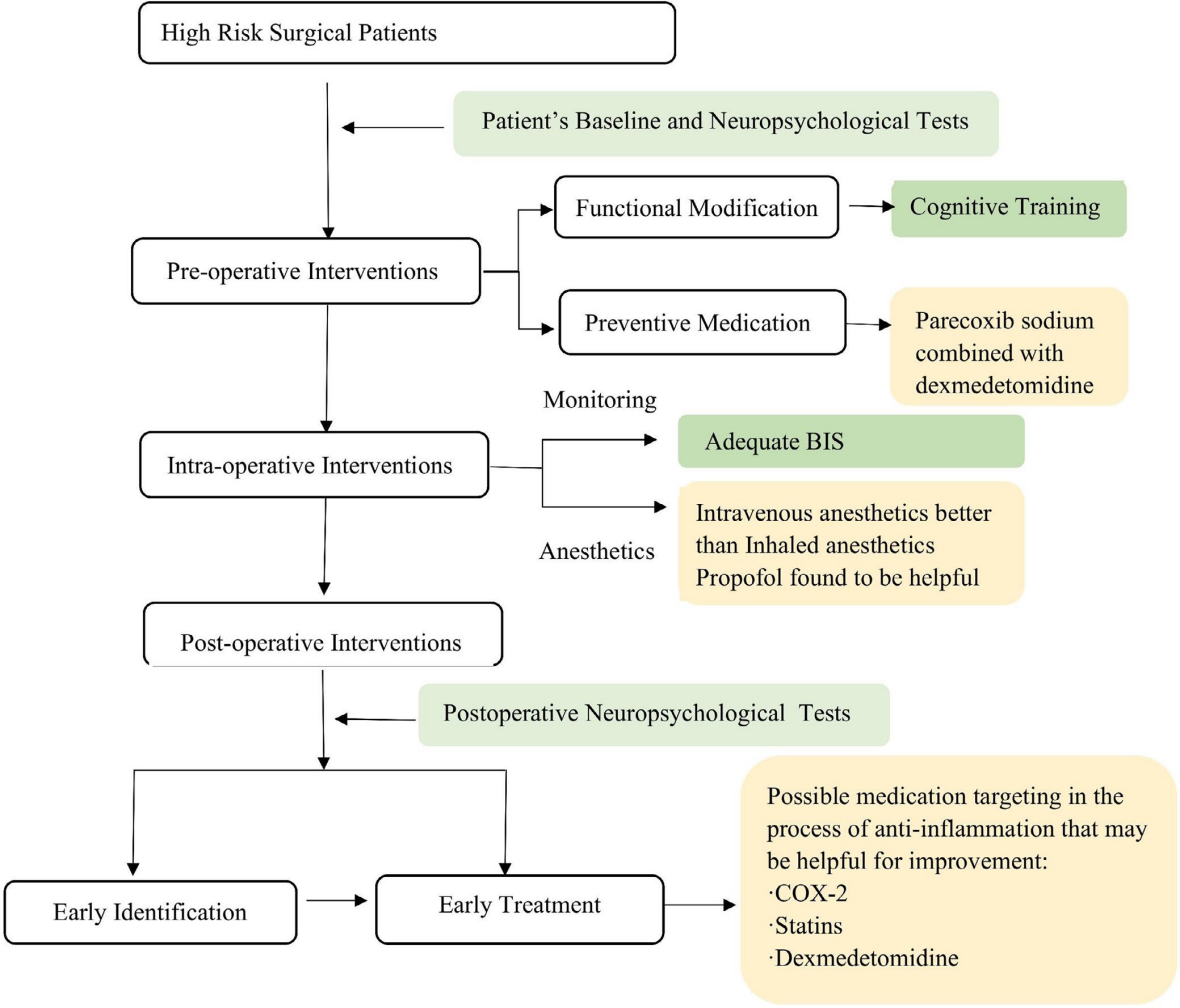
Preventive measures of POCD based on latest research on it during preoperative, intraoperative, and postoperative process and possible effective medications that may contribute to the prevention. POCD, postoperative cognitive dysfunction. BIS, bispectral index. COX‐2, cyclooxygenase‐2

As for monitoring measures, one trial has shown a decreased incidence of short‐term POCD with bispectral index (BIS)‐guided deep anesthesia during the operation.^69^ Lastly, it has been suggested that postoperative management, including early identification and treatment of postoperative complications, may decrease the risk of POCD, which will be discussed in the treatment section. All interventions discussed above are listed in Figure 2.

### 4.3 Prevention of stroke

As for perioperative ischemic (covert) stroke and hemorrhagic stroke, the control of modifiable risk factors is the most effective preventive strategy. These identified modifiable risk factors include hypertension, diabetes mellitus, hyperlipidemia, obesity, and smoking.^70^

#### 4.3.1 Modifiable risk factor—hypertension

Hypertension is the most important modifiable factor for stroke,^71^ attributing to more than half of all stroke events worldwide.^72^ Antihypertensive medications are recommended for patients with BP over 140/90 mmHg.^73^ The most common medications include β‐adrenergic agonists (β‐blockers), calcium channel blockers (CCB), diuretics, angiotensin‐converting enzyme inhibitors (ACEI), and angiotensin II receptors blockers (ARB), and the choice of therapy depends on individual comorbidities.^73^ Adequate BP control is important, while the goal adjustment should also be considered for older patients to avoid complications such as hypotension and dizziness. Although perioperative use of β‐blockers might be beneficial in reducing heart rate and sympathetic activity and controlling BP, there is no association between β‐blockers and perioperative outcomes.^74^, ^75^

#### 4.3.2 Modifiable risk factor—hyperlipidemia

Hyperlipidemia is another remarkable risk factor, as several clinical trials and meta‐analyses have reported decreased vascular events and mortality rates in patients with treatment for hyperlipidemia, especially lowering LDL‐C (low‐density lipoprotein‐cholesterol).^72^ A randomized trial evaluated the benefits of statin as secondary prevention of stroke and found that atorvastatin can reduce the incidence of stroke in patients with recent stroke or TIA.^76^

Treatments of hypertension and hyperlipidemia have been proven to prevent stroke. However, the effectiveness of other factors such as weight and blood glucose control, or smoking cessation may warrant further trials. Besides these control measures against risk factors, there are some other strategies proved to prevent postoperative stroke. Researchers have found that perioperative use of dexmedetomidine reduced the incidence of postoperative stroke and delirium in elderly patients following cardiac surgery.^77^ And in another clinical trial, left atrial appendage (LAA) surgical exclusion is effective on prevention of stroke, particularly in patients with atrial fibrillation after mitral valve replacement (MVR).^55^

## 5 CLINICAL MANAGEMENT AND PRECLINICAL STRATEGIES

Immediate treatment of etiologies and symptoms can contribute to a shorter duration of postoperative neurological disorders and lower risk of long‐term cognitive impairment, disability, and even death.^78^, ^79^ The clinical treatments and preclinical studies of POD, POCD, postoperative ischemic stroke, and postoperative hemorrhagic stroke are discussed below.

### 5.1 Treatment of POD

#### 5.1.1 Nonpharmacological measures

Nonpharmacological measures are beneficial for both preventing and treating POD,^80^ including reorientation, early mobilization, promotion of sleep cycle, adequate hydration, and visual and hearing aids. They can modify and create a safe and calm environment for patients.

#### 5.1.2 Pharmacological measures

There is no strong evidence for pharmacological management of POD, although the use of dexmedetomidine resulted in more ventilator‐free time at 7 days among patients with agitated delirium in the intensive care unit.^81^ Medications are generally applied for delirium‐associated behaviors. Two types of medications are frequently used, that is, antipsychotics and benzodiazepines.^82^

For agitation with perceptual disturbance or sleep‐wake cycle abnormalities, antipsychotics can be useful.^83^ Nevertheless, patients with POD, which is more common in advanced age, may respond poorly to antipsychotic medications.^84^ Benzodiazepines are historically used to sedate patients with delirium for decades. However, evidence suggests that benzodiazepines may increase the risk and duration of delirium, especially in elderly patients.^85^ Thus, benzodiazepines should be mainly used in the treatment of agitation associated with sedative withdrawal.^82^ Another randomized trial showed that the addition of lorazepam to haloperidol resulted in a significantly reduction in agitation at 8 hours in hospitalized cancer patients with agitated delirium.^86^ Given the above evidence, pharmacologic treatment is not strongly recommended but can be used to treat severe agitation and life‐threatening POD complication.

### 5.2 Treatment of POCD

Preventive interventions discussed above can also be used for the treatment of POCD. Several preclinical and clinical studies suggested that targeting postoperative neuro‐inflammation^87^, ^88^, ^89^, ^90^, ^91^ may be a potential way to treat POCD. There are some medications used in the clinical trials attempted to block the process of neuroinflammation.

The cyclooxygenase‐2 (COX‐2), which is responsible for catalyzing the conversion of arachidonic acid to pro‐inflammatory prostaglandins^92^ and increasing blood‐brain barrier (BBB) permeability,^93^, ^94^ is considered to be an important mediator of neuroinflammation and thus a potential target for POCD treatment. Moreover, a meta‐analysis suggested that the administration of parecoxib was effective in treating early POCD within 7 days and reducing interleukin‐6 (IL‐6) and S100 calcium‐binding protein B protein (S100β) concentrations within 2 days after operations.^95^ Other anti‐inflammatory medications, such as minocycline^96^ and dexamethasone,^97^ may also provide possible treatments for POCD. However, no prospective clinical trials investigated the promising effect of antioxidative agents for the prevention of POCD.

Statins are reversible competitive inhibitors of the rate‐limiting enzyme in cholesterol synthesis.^98^ They have been widely proven to be beneficial for neurological disorders, including dementia^99^ and delirium.^100^ In POCD, a clinical trial showed a significant reduction in memory dysfunction comparing statin to placebo in patients undergoing off‐pump coronary artery bypass grafting (CABG).^101^

Dexmedetomidine has downstream effects on reducing serum pro‐inflammatory cytokines in POCD.^102^ Dexmedetomidine treatment has been shown to ameliorate neurological dysfunction and decrease the incidence of cognitive impairment following surgical trauma in a hyperlipidemia rat model.^103^

### 5.3 Treatment of postoperative ischemic stroke

Postoperative covert stroke, as a type of postoperative ischemic stroke, has been studied little so far, and thus, there is no recommended treatment. Focusing on underlying treatable causes may be helpful but it is still in need of further investigation. Further research developed, treatment protocol may be clearer with more information. There are several clinical treatments for stroke, including intravenous thrombolysis, mechanical thrombectomy, intravenous infusion of unfractionated heparin,^104^ which are major treatments for stroke. As for postoperative stroke, some of the treatments may not be suitable for patients undergoing surgeries. For example, intravenous unfractionated heparin is not recommended for postoperative stroke due to the high incidence of bleeding. However, the prolonged therapeutic window and new thrombectomy devices will give more opportunities to treat postoperative ischemic stroke in the future. Several effective treatments of postoperative ischemic stroke are listed below, which may be possible complications of covert stroke.

#### 5.3.1 Intravenous thrombolysis, injecting intravenous medications to dissolve thrombus

For patients with ischemic stroke up to 4.5 hours after symptom onset,^105^ intravenous thrombolysis using tissue‐type plasminogen activator (t‐PA) can be considered as the first‐line treatment. However, undergoing major surgery within 14 days is a contraindication for t‐PA administration because of bleeding of surgical sites. Therefore, it is challenging and controversial to use intravenous t‐PA even with clear symptoms for patients with postoperative stroke.^106^ The use of t‐PA should be individualized based on the risk and benefit of the treatment.

#### 5.3.2 Mechanical thrombectomy, using a guidewire to remove thrombus

Multiple clinical trials have demonstrated that endovascular therapy (EVT) has effective recanalization^107^ and better clinical outcomes without the additional complication of hemorrhage in anterior circulation ischemia with large vessel occlusion (mainly of the intracranial internal carotid artery [ICA], middle cerebral artery [MCA] main trunk, or the M2 major branch of the MCA), which is considered as a treatment option for postoperative stroke.^108^ Besides, studies have expanded the therapeutic window up to 24 hours after symptom onset for patients with a small core and large penumbra.^109^ New devices continue to be developed for reaching distal branches,^110^ which may provide standard treatment for perioperative stroke.

### 5.4 Treatment of postoperative hemorrhagic stroke

Some clinical trials suggested various effective treatment methods for hemorrhagic stroke, including the control of high BP and intracranial pressure (ICP), and treatment of complications and intracranial hemorrhage,^111^ which are important treatment methods for hemorrhagic stroke. We will discuss the most effective methods below.

#### 5.4.1 Controlled BP

Patients with hemorrhage usually present high BP. High SBP has been associated with neurological deterioration and death,^112^ which should be gradually reduced by using antihypertensive drugs such as CCB and ACEI. The ASA recommended that for patients presenting with SBP between 150 and 220 mmHg, an acute and aggressive reduction of SBP is safe and beneficial for functional outcomes.^112^ However, some clinical trials found no significant relationship between SBP reduction and hematoma expansion or outcome.^113^ The goal and effect of BP control in postoperative hemorrhagic stroke need to be elucidated in future studies.

#### 5.4.2 Interventions to control intracranial pressure (ICP)

The initial treatment should include elevating the head of the bed to 30° and the patients’ head facing midline to avoid excessive flexion or rotation of the neck.^114^ Then, osmotic agents (mannitol and hypertonic saline) are given; for example, 20% mannitol is given at 1.0–1.5 g/kg.^115^ Next, early intubation and mechanical ventilation should be applied, especially for patients in coma. ASA also recommends ICP monitoring with a ventricular catheter to ensure the cerebral perfusion pressure (CPP) between 50 and 60 mmHg to prevent cerebral ischemia.^112^

#### 5.4.3 Treatment of complications

Researches showed that 3% to 17% of patients have seizures^116^ in the first two weeks after operation, which should be treated with antiepileptic drugs.^112^ First‐generation antiepileptic drugs negatively affect cognitive function, which should be avoided in patients with postoperative stroke.^117^ Drugs developed after 2000 are known as third‐generation antiepileptic drugs, such as eslicarbazepine acetate, lacosamide, perampanel, brivaracetam, rufinamide, and stiripentol.^118^ These drugs, characterized with new mechanisms of action and favorable pharmacokinetics, can decrease the occurrence of side effects and drug‐drug or hormonal interactions compared with first‐generation antiepileptic drugs. These new drugs are also useful therapies in patients with intractable epilepsy.^119^ However, currently there are no instructions on the use of these drugs for postoperative stroke patients.

#### 5.4.4 Surgical treatments of hemorrhage

There are different types of surgical treatments of hemorrhagic stroke, including craniotomy for hematoma drainage, decompressive craniectomy with or without hematoma drainage, minimally invasive endoscopic aspiration and catheter aspiration.^120^

##### Open surgery

Although the approach of open surgery to treat patients with cerebral hemorrhage remains controversial, it is still one of the most common approaches applied for hematoma drainage.^121^ The Surgical Trial in Intracerebral Hemorrhage (STICH), which was the first multicenter, multinational, randomized clinical trial that compared the benefits of hematoma drainage with conservative management, found that there was no overall benefit from early hematoma drainage compared with conservative treatment.^122^ Subsequently, another international, multicenter, prospective, randomized trial, which included only patients with superficial hematomas within 1 cm from the brain's cortical surface of the brain, indicated that patients with superficial hematomas could benefit from early intervention of hematoma removal.^123^

##### Minimally invasive approach

The practice of open craniotomy is complicated with brain tissue damage and related complications because it requires a large bone flap and exposure of the brain tissue. On the contrary, a minimally invasive approach with thrombolysis is safer, more feasible, and more efficacious.^124^ However, it still showed no significant benefit of long‐term functional outcome than conservative treatment based on clinical trials.^124^

In summary, decompressive craniectomy and hematoma evacuation are performed more frequently for hemorrhagic stroke in patients with Glasgow Coma Scale (GCS) scores of 8 or less and large hematomas. These procedures reduce mortality and may improve functional outcomes.^125^

## 6 CONCLUSION AND FUTURE REMARKS

Postoperative neurological disorders are common complications without effective treatment that exert an enormous burden on patients, their families, hospitals, public resources, and society. The existing acknowledge of risk factors provides certain information to guide possible effective interventions that may need more repeating trials, but further interventional research is urgently needed to improve the outcomes or prognosis for hospitalized patients who experience the condition. Early prevention is likely to be more effective than treatment for prognosis. Thus, further prospective trials should gain deeper insight into uncertain mechanisms and contributing factors underlying these neurological disorders, avoiding simple risk factor evaluation, and validating possible prognostic models for interventional research.

Significantly, dexmedetomidine may be an effective medication for the prevention of POD and POCD as mentioned in multiple trials, which should be considered in future research. As for covert stroke, early identification may be more effective, likely given priority to in further research.

## CONFLICT OF INTEREST

The authors declare to have no potential conflicts of interest. All data included in this study are available upon request by contact with the corresponding author.

## References

[1] Haenggi M , Blum S , Brechbuehl R , Brunello A , Jakob SM , Takala J . Effect of sedation level on the prevalence of delirium when assessed with CAM‐ICU and ICDSC. Intensive Care Med. 2013;39:2171‐2179. 23921976 10.1007/s00134-013-3034-5

[2] Sharma PT , Sieber FE , Zakriya KJ , et al. Recovery room delirium predicts postoperative delirium after hip‐fracture repair. Anest Analg. 2005;101:1215‐1220. 10.1213/01.ane.0000167383.44984.e5 16192548

[3] Wang P , Velagapudi R , Kong C , et al. Neurovascular and immune mechanisms that regulate postoperative delirium superimposed on dementia. Alzheimer's & Dementia. 2020;16:734‐749. 10.1002/alz.12064 PMC7317948 32291962

[4] Sachdev PS , Blacker D , Blazer DG , et al. Classifying neurocognitive disorders: the DSM‐5 approach. Nat Rev Neurol. 2014;10:634‐642. 25266297 10.1038/nrneurol.2014.181

[5] Shenkin SD , Fox C , Godfrey M , et al. Delirium detection in older acute medical inpatients: a multicentre prospective comparative diagnostic test accuracy study of the 4AT and the confusion assessment method. BMC Med. 2019;17:138. 31337404 10.1186/s12916-019-1367-9 PMC6651960

[6] Kotfis K , Marra A , Ely EW . ICU delirium — A diagnostic and therapeutic challenge in the intensive care unit. Anaesthesiology Intensive. Therapy. 2018;50. 10.5603/AIT.a2018.0011 29882581

[7] Borchers F , Knaak C , Piper SK , Spies C . Empfehlungen zur Erfassung und Beschreibung perioperativer kognitiver Störungen in Wissenschaft und Praxis. Anästhesiol Intensivmed Notfallmed Schmerzther. 2019;54:652‐667. 31805585 10.1055/a-0853-3060

[8] Le Y , Liu S , Peng M , et al. Aging differentially affects the loss of neuronal dendritic spine, neuroinflammation and memory impairment at rats after surgery. PLoS One. 2014;9:e106837. 25198176 10.1371/journal.pone.0106837 PMC4157839

[9] Berger M , Terrando N , Smith SK , Browndyke JN , Newman MF , Mathew JP . Neurocognitive Function after Cardiac Surgery: From Phenotypes to Mechanisms. Anesthesiology. 2018;129:829‐851. 29621031 10.1097/ALN.0000000000002194 PMC6148379

[10] Daiello LA , Racine AM , Yun Gou R , et al. Postoperative delirium and postoperative cognitive dysfunction: overlap and divergence. Anesthesiology. 2019;131:477‐491. 31166241 10.1097/ALN.0000000000002729 PMC6692220

[11] Krenk L , Rasmussen LS , Kehlet H . New insights into the pathophysiology of postoperative cognitive dysfunction. Acta Anaesthesiol Scand. 2010;54:951‐956. 20626359 10.1111/j.1399-6576.2010.02268.x

[12] Moller JT , Cluitmans P , Rasmussen LS , et al. Long‐term postoperative cognitive dysfunction in the elderly: ISPOCD1 study. Lancet. 1998;351:857‐861. 9525362 10.1016/s0140-6736(97)07382-0

[13] Olotu C . Postoperative neurocognitive disorders. Curr Opin Anaesthesiol. 2020;33:101‐108. 31764008 10.1097/ACO.0000000000000812

[14] Mathers CD , Loncar D . Projections of global mortality and burden of disease from 2002 to 2030. PLoS Medicine. 2006;3:e442. 17132052 10.1371/journal.pmed.0030442 PMC1664601

[15] Zhou M , Wang H , Zeng X , et al. Mortality, morbidity, and risk factors in China and its provinces, 1990–2017: A systematic analysis for the global burden of disease study 2017. The Lancet. 2019;394:1145‐1158. 10.1016/S0140-6736(19)30427-1 PMC6891889 31248666

[16] Dong Y , Cao W , Cheng X , et al. Risk factors and stroke characteristic in patients with postoperative strokes. J Stroke Cerebrovasc Dis. 2017;26:1635‐1640. 28478979 10.1016/j.jstrokecerebrovasdis.2016.12.017

[17] Vlisides PE , Avidan MS , Mashour GA . Uncovering covert stroke in surgical patients. The Lancet. 2019;394:982‐984. 10.1016/S0140-6736(19)31770-2 31422896

[18] Chen H‐F , Huang L‐L , Li H‐Y , et al. Microstructural disruption of the right inferior fronto‐occipital and inferior longitudinal fasciculus contributes to WMH‐related cognitive impairment. CNS Neurosci Ther. 2020;26:576‐588. 31901155 10.1111/cns.13283 PMC7163793

[19] Yu S , Li P . Cognitive declines after perioperative covert stroke: Recent advances and perspectives. Curr Opin Anaesthesiol. 2020;33:651‐654. 32796168 10.1097/ACO.0000000000000903

[20] Lukaszewicz AC , Bouchier B , Bruckert V . Perioperative covert stroke: An overlooked but sneaky event. Anaesth Crit Care Pain Med. 2020;39:19‐20. 31874227 10.1016/j.accpm.2019.12.003

[21] Mrkobrada M , Hill MD , Chan MTV , et al. Covert stroke after non‐cardiac surgery: A prospective cohort study. Br J Anaesth. 2016;117:191‐197. 27440630 10.1093/bja/aew179

[22] Mrkobrada M , Chan MTV , Cowan D , et al. Perioperative covert stroke in patients undergoing non‐cardiac surgery (NeuroVISION): A prospective cohort study. The Lancet. 2019;394:1022‐1029. 10.1016/S0140-6736(19)31795-7 31422895

[23] Montaño A , Hanley DF , Hemphill JC . Chapter 13 ‐ Hemorrhagic stroke. In: Hetts SW , Cooke DL , eds. Handbook of clinical neurology. Elsevier; 2021:229‐248. 10.1016/B978-0-444-64034-5.00019-5 33272397

[24] Virani SS , Alonso A , Benjamin EJ , et al. Heart disease and stroke statistics—2020 update: A report from the american heart association. Circulation. 2020;141:e139‐e596. 31992061 10.1161/CIR.0000000000000757

[25] Selim M , Hanley D , Steiner T , et al. Recommendations for clinical trials in ICH. Stroke. 2020;51:1333‐1338. 32078490 10.1161/STROKEAHA.119.027882 PMC7093252

[26] Kameyama M , Fujimura M , Tashiro R , et al. Significance of quantitative cerebral blood flow measurement in the acute stage after revascularization surgery for adult moyamoya disease: Implication for the pathological threshold of local cerebral hyperperfusion. Cerebrovasc Dis. 2019;48:217‐225. 31812964 10.1159/000504835

[27] Dong H , Liu S , Jing L , et al. Hypertension among hemorrhagic stroke patients in northeast China: A population‐based study 2017–2019. Med Sci Monit. 2020;26:e926581. 33376232 10.12659/MSM.926581 PMC7781047

[28] Ranasinghe JS , Kafi S , Oppenheimer J , Birnbach DJ . Hemorrhagic stroke following elective cesarean delivery. Int J Obstet Anesth. 2008;17:271‐274. 18511257 10.1016/j.ijoa.2007.10.005

[29] van Mook WNKA , Rennenberg RJMW , Schurink GW , et al. Cerebral hyperperfusion syndrome. Lancet Neurol. 2005;4:877‐888. 16297845 10.1016/S1474-4422(05)70251-9

[30] Lin X , Chen Y , Zhang P , Chen G , Zhou Y , Yu X . The potential mechanism of postoperative cognitive dysfunction in older people. Exp Gerontol. 2020;130: 110791. 10.1016/j.exger.2019.110791 31765741

[31] Abad‐Gurumeta A , Casans‐Frances R , Gomez‐Rios MA . Postoperative neurocognitive disorders: unknowns to solve and work to do. Minerva Anestesiol. 2020;86:908‐909. 32613815 10.23736/S0375-9393.20.14796-5

[32] Aldecoa C , Bettelli G , Bilotta F , et al. European Society of Anaesthesiology evidence‐based and consensus‐based guideline on postoperative delirium. Eur J Anaesthesiol. 2017;34:192‐214. 28187050 10.1097/EJA.0000000000000594

[33] Pandharipande P , Shintani A , Peterson J , et al. Lorazepam is an independent risk factor for transitioning to delirium in intensive care unit patients. Anesthesiology. 2006;104:21‐26. 16394685 10.1097/00000542-200601000-00005

[34] Kapoor I , Prabhakar H , Mahajan C . Postoperative cognitive dysfunction. Indian J Crit Care Med. 2019;23:S162‐S164. 31485127 10.5005/jp-journals-10071-23196 PMC6707501

[35] Baig AM . Neurological manifestations in COVID‐19 caused by SARS‐CoV‐2. CNS Neurosci Ther. 2020;26:499‐501. 32266761 10.1111/cns.13372 PMC7163592

[36] Wei P , Lyu W , Wan T , et al. COVID‐19: a novel risk factor for perioperative neurocognitive disorders. Br J Anaesth. 2021;127:e113‐e115. 34266660 10.1016/j.bja.2021.06.016 PMC8214172

[37] Tsiouris A , Heliopoulos I , Mikroulis D , Mitsias PD . Factors defining occurrence of ischemic and hemorrhagic strokes during continuous flow left ventricular assist device support. Gen Thorac Cardiovasc Surg. 2020;68:319‐327. 31435873 10.1007/s11748-019-01190-8

[38] Xie N , Meng X , Wu C , et al. Both left upper lobectomy and left pneumonectomy are risk factors for postoperative stroke. Sci Rep 2019;9:10432. 31320706 10.1038/s41598-019-46989-w PMC6639360

[39] Lin M‐H , Kamel H , Singer DE , Wu Y‐L , Lee M , Ovbiagele B . Perioperative/postoperative atrial fibrillation and risk of subsequent stroke and/or mortality. Stroke. 2019;50:1364‐1371. 31043148 10.1161/STROKEAHA.118.023921

[40] Shin YS . Postoperative delirium in geriatric patients after emergency general surgery. J Am Coll Surg. 2020;231:188‐189. 32414684 10.1016/j.jamcollsurg.2020.04.018

[41] Ko S‐B . Perioperative stroke: pathophysiology and management. Korean J Anesthesiol. 2018;71:3‐11. 29441169 10.4097/kjae.2018.71.1.3 PMC5809704

[42] Al‐Qamari A , Adeleke I , Kretzer A , Hogue CW . Pulse pressure and perioperative stroke. Curr Opin Anaesthesiol. 2019;32:57‐63. 30543556 10.1097/ACO.0000000000000673 PMC6310080

[43] Khanna AK , Shaw AD , Stapelfeldt WH , et al. Postoperative hypotension and adverse clinical outcomes in patients without intraoperative hypotension, after noncardiac surgery. Anesth Analg. 2021;132:1410‐1420. 33626028 10.1213/ANE.0000000000005374

[44] Gregory A , Stapelfeldt WH , Khanna AK , et al. Intraoperative hypotension is associated with adverse clinical outcomes after noncardiac surgery. Anesth Analg. 2021;132:1654‐1665. 33177322 10.1213/ANE.0000000000005250 PMC8115733

[45] Yu Q , Qi J , Wang Y . Intraoperative hypotension and neurological outcomes. Curr Opin Anaesthesiol. 2020;33:646‐650. 32769747 10.1097/ACO.0000000000000904

[46] Bo M , Marchionni N . Practical use of direct oral anti coagulants (DOACs) in the older persons with atrial fibrillation. Eur J Intern Med. 2020;71:32‐38. 31740104 10.1016/j.ejim.2019.10.026

[47] Leonidas P , Jeremy M , Damianos GK , et al. Reversal of novel anticoagulants in emergent surgery and trauma: A comprehensive review and proposed management algorithm. Curr Pharm Des. 2018;24:4540‐4553. 30585542 10.2174/1381612825666181226150629

[48] Kuramatsu JB , Sembill JA , Huttner HB . Reversal of oral anticoagulation in patients with acute intracerebral hemorrhage. Crit Care. 2019;23:206. 31171018 10.1186/s13054-019-2492-8 PMC6555738

[49] Adeboyeje G , Sylwestrzak G , Barron JJ , et al. Major bleeding risk during anticoagulation with warfarin, dabigatran, apixaban, or rivaroxaban in patients with nonvalvular atrial fibrillation. J Manag Care Spec Pharm. 2017;23:968‐978. 28854073 10.18553/jmcp.2017.23.9.968 PMC10398327

[50] Milling TJ Jr , Ziebell CM . A review of oral anticoagulants, old and new, in major bleeding and the need for urgent surgery. Trends Cardiovasc Med. 2020;30:86‐90. 30952383 10.1016/j.tcm.2019.03.004 PMC6763385

[51] Lovell N , Maddocks M , Etkind SN , et al. Characteristics, symptom management, and outcomes of 101 patients With COVID‐19 referred for hospital palliative care. J Pain Symptom Manage. 2020;60:e77‐e81. 32325167 10.1016/j.jpainsymman.2020.04.015 PMC7169932

[52] Singler K , Thomas C . HELP – Hospital Elder Life Program – ein multimodales Interventionsprogramm zur Delirprävention bei älteren Patienten. Der Internist. 2017;58:125‐131. 28120023 10.1007/s00108-016-0181-0

[53] Abraha I , Trotta F , Rimland JM , et al. Efficacy of non‐pharmacological interventions to prevent and treat delirium in older patients: A systematic overview. The SENATOR project ONTOP series. PLoS One. 2015;10:e0123090. 26062023 10.1371/journal.pone.0123090 PMC4465742

[54] Deng LX , Cao L , Zhang LN , Peng XB , Zhang L . Non‐pharmacological interventions to reduce the incidence and duration of delirium in critically ill patients: A systematic review and network meta‐analysis. J Crit Care. 2020;60:241‐248. 32919363 10.1016/j.jcrc.2020.08.019

[55] Jiang S , Zhang H , Wei S , et al. Left atrial appendage exclusion is effective in reducing postoperative stroke after mitral valve replacement. J Card Surg. 2020;35:3395‐3402. 32939788 10.1111/jocs.15020

[56] Wildes TS , Mickle AM , Ben Abdallah A , et al. Effect of electroencephalography‐guided anesthetic administration on postoperative delirium among older adults undergoing major surgery: The ENGAGES randomized clinical trial. JAMA. 2019;321:473‐483. 30721296 10.1001/jama.2018.22005 PMC6439616

[57] Wu Y‐C , Tseng P‐T , Tu Y‐K , et al. Association of delirium response and safety of pharmacological interventions for the management and prevention of delirium: A network meta‐analysis. JAMA Psychiatry. 2019;76:526‐535. 30810723 10.1001/jamapsychiatry.2018.4365 PMC6495351

[58] Sun Y , Lin D , Wang J , et al. Effect of tropisetron on prevention of emergence delirium in patients after noncardiac surgery: A trial protocol. JAMA Netw Open 2020;3:e2013443. 33052400 10.1001/jamanetworkopen.2020.13443 PMC7557499

[59] Subramaniam B , Shankar P , Shaefi S , et al. Effect of intravenous acetaminophen vs placebo combined with propofol or dexmedetomidine on postoperative delirium among older patients following cardiac surgery: The DEXACET randomized clinical trial. JAMA. 2019;321:686‐696. 30778597 10.1001/jama.2019.0234 PMC6439609

[60] Siddiqi N , Harrison JK , Clegg A , et al. Interventions for preventing delirium in hospitalised non‐ICU patients. Cochrane Database of Systematic Reviews. 2016;3:CD005563. 10.1002/14651858.CD005563.pub3 PMC10431752 26967259

[61] Mistraletti G , Umbrello M , Anania S , et al. Neurological assessment with validated tools in general ICU: multicenter, randomized, before and after, pragmatic study to evaluate the effectiveness of an e‐learning platform for continuous medical education. Minerva Anestesiol. 2017;83:145‐154. 27647465 10.23736/S0375-9393.16.11103-4

[62] Rasmussen LS , Larsen K , Houx P , et al. The assessment of postoperative cognitive function. Acta Anaesthesiol Scand. 2001;45:275‐289. 11207462 10.1034/j.1399-6576.2001.045003275.x

[63] Bliss ES , Wong RH , Howe PR , Mills DE . Benefits of exercise training on cerebrovascular and cognitive function in ageing. J Cereb Blood Flow Metab. 2021;41:447‐470. 32954902 10.1177/0271678X20957807 PMC7907999

[64] O'Gara BP , Mueller A , Gasangwa DVI , et al. Prevention of early postoperative decline: A randomized, controlled feasibility trial of perioperative cognitive training. Anesth Analg. 2020;130:586‐595. 31569161 10.1213/ANE.0000000000004469 PMC7154961

[65] Lu J , Chen G , Zhou H , Zhou Q , Zhu Z , Wu C . Effect of parecoxib sodium pretreatment combined with dexmedetomidine on early postoperative cognitive dysfunction in elderly patients after shoulder arthroscopy: A randomized double blinded controlled trial. J Clin Anesth. 2017;41:30‐34. 28802598 10.1016/j.jclinane.2017.06.004 PMC5558812

[66] Berndt N , Kovács R , Schoknecht K , et al. Low neuronal metabolism during isoflurane‐induced burst suppression is related to synaptic inhibition while neurovascular coupling and mitochondrial function remain intact. J Cereb Blood Flow Metab. 0:0271678X211010353. 10.1177/0271678X211010353 PMC8504408 33899556

[67] Chen F , Duan G , Wu Z , Zuo Z , Li H . Comparison of the cerebroprotective effect of inhalation anaesthesia and total intravenous anaesthesia in patients undergoing cardiac surgery with cardiopulmonary bypass: a systematic review and meta‐analysis. BMJ Open. 2017;7:e014629. 10.1136/bmjopen-2016-014629 PMC5652618 29025825

[68] Li W‐X , Luo R‐Y , Chen C , et al. Effects of propofol, dexmedetomidine, and midazolam on postoperative cognitive dysfunction in elderly patients: a randomized controlled preliminary trial. Chin Med J. 2019;132:437‐445. 30707179 10.1097/CM9.0000000000000098 PMC6595716

[69] Quan C , Chen J , Luo Y , et al. BIS‐guided deep anesthesia decreases short‐term postoperative cognitive dysfunction and peripheral inflammation in elderly patients undergoing abdominal surgery. Brain and Behavior. 2019;9:e01238. 30815998 10.1002/brb3.1238 PMC6456817

[70] Udesh R , Mehta A , Gleason TG , Wechsler L , Thirumala PD . Perioperative strokes and early outcomes in mitral valve surgery: A nationwide analysis. J Cardiothorac Vasc Anesth. 2017;31:529‐536. 28259602 10.1053/j.jvca.2016.12.006

[71] Cipolla MJ , Liebeskind DS , Chan S‐L . The importance of comorbidities in ischemic stroke: Impact of hypertension on the cerebral circulation. J Cereb Blood Flow Metab. 2018;38:2129‐2149. 30198826 10.1177/0271678X18800589 PMC6282213

[72] O'Donnell MJ , Chin SL , Rangarajan S , et al. Global and regional effects of potentially modifiable risk factors associated with acute stroke in 32 countries (INTERSTROKE): A case‐control study. The Lancet. 2016;388:761‐775. 10.1016/S0140-6736(16)30506-2 27431356

[73] Malhotra K , Goyal N , Katsanos AH , et al. Association of blood pressure with outcomes in acute stroke thrombectomy. Hypertension. 2020;75:730‐739. 31928111 10.1161/HYPERTENSIONAHA.119.14230 PMC7233454

[74] van Lier F , Schouten O , Hoeks SE , et al. Impact of prophylactic beta‐blocker therapy to prevent stroke after noncardiac surgery. Am J Cardiol. 2010;105:43‐47. 20102888 10.1016/j.amjcard.2009.08.646

[75] Hajibandeh S , Hajibandeh S , Antoniou SA , Torella F , Antoniou GA . Effect of beta‐blockers on perioperative outcomes in vascular and endovascular surgery: A systematic review and meta‐analysis. Br J Anaesth. 2017;118:11‐21. 28039238 10.1093/bja/aew380

[76] Szarek M , Amarenco P , Callahan A , et al. Atorvastatin reduces first and subsequent vascular events across vascular territories: The SPARCL trial. J Am Coll Cardiol. 2020;75:2110‐2118. 32194196 10.1016/j.jacc.2020.03.015

[77] Cheng H , Li Z , Young N , et al. The effect of dexmedetomidine on outcomes of cardiac surgery in elderly patients. J Cardiothorac Vasc Anesth. 2016;30:1502‐1508. 27435836 10.1053/j.jvca.2016.02.026 PMC5010787

[78] Adults TAGSEPoPDiO . American geriatrics society abstracted clinical practice guideline for postoperative delirium in older adults. J Am Geriatr Soc. 2015;63:142‐150. 25495432 10.1111/jgs.13281 PMC5901697

[79] Young J , Murthy L , Westby M , Akunne A , O’Mahony R . Diagnosis, prevention, and management of delirium: Summary of NICE guidance. BMJ. 2010;341:c3704. 20667955 10.1136/bmj.c3704

[80] Yürek F , Olbert M , Müller‐Werdan U , et al. Wie können postoperativ ein Delir und eine neurokognitive Störung verhindert werden? Anästhesiol Intensivmed Notfallmed Schmerzther. 2019;54:669‐683. 31805586 10.1055/a-0853-3116

[81] Reade MC , Eastwood GM , Bellomo R , et al. Effect of dexmedetomidine added to standard care on ventilator‐free time in patients with agitated delirium: A randomized clinical trial. JAMA. 2016;315:1460‐1468. 26975647 10.1001/jama.2016.2707

[82] Lauretani F , Bellelli G , Pelà G , Morganti S , Tagliaferri S , Maggio M . Treatment of delirium in older persons: What we should not do! Int J Mol Sci. 2020;21:2397. 10.3390/ijms21072397 PMC7177924 32244301

[83] Aringhieri S , Carli M , Kolachalam S , et al. Molecular targets of atypical antipsychotics: From mechanism of action to clinical differences. Pharmacol Ther. 2018;192:20‐41. 29953902 10.1016/j.pharmthera.2018.06.012

[84] Janssen TL , Alberts AR , Hooft L , Mattace‐Raso F , Mosk CA , van der Laan L . Prevention of postoperative delirium in elderly patients planned for elective surgery: Systematic review and meta‐analysis. Clin Interv Aging. 2019;14:1095‐1117. 31354253 10.2147/CIA.S201323 PMC6590846

[85] Weinstein SM , Poultsides L , Baaklini LR , et al. Postoperative delirium in total knee and hip arthroplasty patients: a study of perioperative modifiable risk factors. Br J Anaesth. 2018;120:999‐1008. 29661417 10.1016/j.bja.2017.12.046

[86] Hui D , Frisbee‐Hume S , Wilson A , et al. Effect of lorazepam with haloperidol vs haloperidol alone on agitated delirium in patients with advanced cancer receiving palliative care: A randomized clinical trial. JAMA. 2017;318:1047‐1056. 28975307 10.1001/jama.2017.11468 PMC5661867

[87] Gong M , Wang G , Li G , et al. Dysfunction of inflammation‐resolving pathways is associated with postoperative cognitive decline in elderly mice. Behav Brain Res. 2020;386:112538. 32113876 10.1016/j.bbr.2020.112538

[88] VanDusen KW , Eleswarpu S , Moretti EW , et al. The MARBLE study protocol: Modulating ApoE signaling to reduce brain inflammation, DeLirium, and PostopErative cognitive dysfunction. J Alzheimers Dis. 2020;75:1319‐1328. 32417770 10.3233/JAD-191185 PMC7923142

[89] Ye J‐S , Chen L , Lu Y‐Y , Lei S‐Q , Peng M , Xia Z‐Y . SIRT3 activator honokiol ameliorates surgery/anesthesia‐induced cognitive decline in mice through anti‐oxidative stress and anti‐inflammatory in hippocampus. CNS Neurosci Ther. 2019;25:355‐366. 30296006 10.1111/cns.13053 PMC6488903

[90] Du S‐Q , Wang X‐R , Zhu W , et al. Acupuncture inhibits TXNIP‐associated oxidative stress and inflammation to attenuate cognitive impairment in vascular dementia rats. CNS Neurosci Ther. 2018;24:39‐46. 29110407 10.1111/cns.12773 PMC6489958

[91] Raz L , Knoefel J , Bhaskar K . The neuropathology and cerebrovascular mechanisms of dementia. J Cereb Blood Flow Metab. 2016;36:172‐186. 26174330 10.1038/jcbfm.2015.164 PMC4758551

[92] Engblom D , Ek M , Saha S , Ericsson‐Dahlstrand A , Jakobsson P‐J , Blomqvist A . Prostaglandins as inflammatory messengers across the blood‐brain barrier. J Mol Med. 2002;80:5‐15. 11862319 10.1007/s00109-001-0289-z

[93] Terrando N , Eriksson LI , Ryu JK , et al. Resolving postoperative neuroinflammation and cognitive decline. Ann Neurol. 2011;70:986‐995. 22190370 10.1002/ana.22664 PMC4556354

[94] Huang X , Hussain B , Chang J . Peripheral inflammation and blood‐brain barrier disruption: Effects and mechanisms. CNS Neurosci Ther. 2021;27:36‐47. 33381913 10.1111/cns.13569 PMC7804893

[95] Huang S , Hu H , Cai Y‐H , Hua F . Effect of parecoxib in the treatment of postoperative cognitive dysfunction: A systematic review and meta‐analysis. Medicine (Baltimore) 2019;98:e13812. 30608392 10.1097/MD.0000000000013812 PMC6344118

[96] Fan L , Wang TL , Xu YC , Ma YH , Ye WG . Minocycline may be useful to prevent/treat postoperative cognitive decline in elderly patients. Med Hypotheses. 2011;76:733‐736. 21354710 10.1016/j.mehy.2011.02.010

[97] Karaman T , Karaman S , Doğru S , Tapar H , Şahin A , Süren M . Short‐term and long‐term effects of dexamethasone on cognitive dysfunction induced by sevoflurane in adult rats. Turk J Anaesthesiol Reanim. 2017;45:158‐163. 28752006 10.5152/TJAR.2017.98624 PMC5512394

[98] Cerqueira N , Oliveira E , Gesto D , et al. Cholesterol biosynthesis: A mechanistic overview. Biochemistry. 2016;55:5483‐5506. 27604037 10.1021/acs.biochem.6b00342

[99] Lee J‐W , Choi E‐A , Kim Y‐S , et al. Statin exposure and the risk of dementia in individuals with hypercholesterolaemia. J Intern Med. 2020;288:689‐698. 32583471 10.1111/joim.13134

[100] Junhui C , Yuhai W , Ximin H , et al. The Role of Statins in the Management of Delirium: Recent Advances. CNS Neurol Disord Drug Targets. 2021;20:203‐215. 32691721 10.2174/1871527319666200720111318

[101] Das S , Nanda SK , Bisoi AK , Wadhawan AN . Effect of preoperative statin therapy on early postoperative memory impairment after off‐pump coronary artery bypass surgery. Ann Card Anaesth. 2016;19:38‐44. 26750672 10.4103/0971-9784.173018 PMC4900397

[102] Li Y , He R , Chen S , Qu Y . Effect of dexmedetomidine on early postoperative cognitive dysfunction and peri‐operative inflammation in elderly patients undergoing laparoscopic cholecystectomy. Exp Ther Med. 2015;10:1635‐1642. 26640530 10.3892/etm.2015.2726 PMC4665836

[103] Zhang XP , Liu YR , Chai M , et al. High‐fat treatment prevents postoperative cognitive dysfunction in a hyperlipidemia model by protecting the blood‐brain barrier via Mfsd2a‐related signaling. Mol Med Rep. 2019;20:4226‐4234. 31545471 10.3892/mmr.2019.10675 PMC6797931

[104] Rabinstein A . Update on treatment of acute ischemic stroke. Continuum (Minneapolis, Minn). 2020;26:268‐286. 10.1212/CON.0000000000000840 32224752

[105] Emberson J , Lees KR , Lyden P , et al. Effect of treatment delay, age, and stroke severity on the effects of intravenous thrombolysis with alteplase for acute ischaemic stroke: A meta‐analysis of individual patient data from randomised trials. Lancet. 2014;384:1929‐1935. 25106063 10.1016/S0140-6736(14)60584-5 PMC4441266

[106] Tekle WG , Hassan AE , Jadhav AP , et al. Impact of periprocedural and technical factors and patient characteristics on revascularization and outcome in the DAWN trial. Stroke. 2020;51:247‐253. 31744425 10.1161/STROKEAHA.119.026437

[107] Laredo C , Renú A , Tudela R , et al. The accuracy of ischemic core perfusion thresholds varies according to time to recanalization in stroke patients treated with mechanical thrombectomy: A comprehensive whole‐brain computed tomography perfusion study. J Cereb Blood Flow Metab. 2020;40:966‐977. 31208242 10.1177/0271678X19855885 PMC7181085

[108] Smith WS . Endovascular stroke therapy. Neurotherapeutics. 2019;16:360‐368. 30838523 10.1007/s13311-019-00724-5 PMC6554365

[109] Nogueira RG , Jadhav AP , Haussen DC , et al. Thrombectomy 6 to 24 hours after stroke with a mismatch between deficit and infarct. N Engl J Med. 2018;378:11‐21. 29129157 10.1056/NEJMoa1706442

[110] Kara B , Selcuk HH , Erbahceci Salik A , et al. Single‐center experience with the Tigertriever device for the recanalization of large vessel occlusions in acute ischemic stroke. J NeuroIntervent Surg. 2019;11:455‐459. 10.1136/neurintsurg-2018-014196 30262656

[111] Lapchak PA , Araujo DM . Advances in hemorrhagic stroke therapy: Conventional and novel approaches. Expert Opin Emerg Drugs. 2007;12:389‐406. 17874968 10.1517/14728214.12.3.389

[112] Hemphill JC , Greenberg SM , Anderson CS , et al. Guidelines for the management of spontaneous intracerebral hemorrhage. Stroke. 2015;46:2032‐2060. 26022637 10.1161/STR.0000000000000069

[113] Qureshi AI , Palesch YY , Martin R , et al. Effect of systolic blood pressure reduction on hematoma expansion, perihematomal edema, and 3‐month outcome among patients with intracerebral hemorrhage: results from the antihypertensive treatment of acute cerebral hemorrhage study. Arch Neurol. 2010;67:570‐576. 20457956 10.1001/archneurol.2010.61 PMC5562043

[114] Schizodimos T , Soulountsi V , Iasonidou C , Kapravelos N . An overview of management of intracranial hypertension in the intensive care unit. J Anesth. 2020;34:741‐757. 32440802 10.1007/s00540-020-02795-7 PMC7241587

[115] Witherspoon B , Ashby NE . The use of mannitol and hypertonic saline therapies in patients with elevated intracranial pressure: A review of the evidence. Nurs Clin North Am. 2017;52:249‐260. 28478873 10.1016/j.cnur.2017.01.002

[116] Fukuma K , Kajimoto K , Tanaka T , et al. Visualizing prolonged hyperperfusion in post‐stroke epilepsy using postictal subtraction SPECT. J Cereb Blood Flow Metab. 2021;41:146‐156. 32065077 10.1177/0271678X20902742 PMC7747161

[117] Beghi E , Beghi M . Epilepsy, antiepileptic drugs and dementia. Curr Opin Neurol. 2020;33:191‐197. 32073437 10.1097/WCO.0000000000000802

[118] Stefanovic S , Jankovic SM , Novakovic M , Milosavljevic M , Folic M . Pharmacodynamics and common drug‐drug interactions of the third‐generation antiepileptic drugs. Expert Opin Drug Metab Toxicol. 2018;14:153‐159. 29268032 10.1080/17425255.2018.1421172

[119] Demir M , Akarsu EO , Dede HO , et al. Investigation of the roles of new antiepileptic drugs and serum BDNF levels in efficacy and safety monitoring and quality of life: A clinical research. Curr Clin Pharmacol. 2020;15:49‐63. 30864528 10.2174/1574884714666190312145409 PMC7497568

[120] Chen S , Zeng L , Hu Z . Progressing haemorrhagic stroke: categories, causes, mechanisms and managements. J Neurol. 2014;261:2061‐2078. 24595959 10.1007/s00415-014-7291-1 PMC4221651

[121] Babi M‐A , James ML . Spontaneous intracerebral hemorrhage: Should we operate? Front Neurol. 2017;8:645. 29375460 10.3389/fneur.2017.00645 PMC5768984

[122] Mendelow AD , Gregson BA , Fernandes HM , et al. Early surgery versus initial conservative treatment in patients with spontaneous supratentorial intracerebral haematomas in the International Surgical Trial in Intracerebral Haemorrhage (STICH): A randomised trial. Lancet. 2005;365:387‐397. 15680453 10.1016/S0140-6736(05)17826-X

[123] Mendelow AD , Gregson BA , Rowan EN , Murray GD , Gholkar A , Mitchell PM . Early surgery versus initial conservative treatment in patients with spontaneous supratentorial lobar intracerebral haematomas (STICH II): a randomised trial. Lancet. 2013;382:397‐408. 23726393 10.1016/S0140-6736(13)60986-1 PMC3906609

[124] Fam MD , Hanley D , Stadnik A , et al. Surgical performance in minimally invasive surgery plus recombinant tissue plasminogen activator for intracerebral hemorrhage evacuation phase III clinical trial. Neurosurgery. 2017;81:860‐866. 28402516 10.1093/neuros/nyx123 PMC6257031

[125] Takeuchi S , Wada K , Nagatani K , Otani N , Mori K . Decompressive hemicraniectomy for spontaneous intracerebral hemorrhage. Neurosurg Focus. 2013;34:E5. 10.3171/2013.2.FOCUS12424 23634924

[126] Wu J , Yin Y , Jin M , Li B . The risk factors for postoperative delirium in adult patients after hip fracture surgery: a systematic review and meta‐analysis. Int J Geriatr Psychiatry. 2021;36:3‐14. 32833302 10.1002/gps.5408

[127] Chan MTV , Cheng BCP , Lee TMC , Gin T . Group tCT. BIS‐guided anesthesia decreases postoperative delirium and cognitive decline. J Neurosurg Anesthesiol. 2013;25:33‐42. 23027226 10.1097/ANA.0b013e3182712fba

[128] Wang LH , Xu DJ , Wei XJ , Chang HT , Xu GH . Electrolyte disorders and aging: risk factors for delirium in patients undergoing orthopedic surgeries. BMC Psychiatry. 2016;16:418. 27881118 10.1186/s12888-016-1130-0 PMC5120472

[129] Robinson TN , Kovar A , Carmichael H , Overbey DM , Goode CM , Jones TS . Postoperative delirium is associated with decreased recovery of ambulation one‐month after surgery. Am J Surg. 2021;221:856‐861. 32933746 10.1016/j.amjsurg.2020.08.031 PMC7910322

[130] Liu J , Yang F , Luo S , et al. Incidence, predictors and outcomes of delirium in complicated type B aortic dissection patients after thoracic endovascular aortic repair. Clin Interv Aging. 2021;16:1581‐1589. 34471348 10.2147/CIA.S328657 PMC8405167

[131] Zhang Y , Bao H‐G , Lv Y‐L , et al. Risk factors for early postoperative cognitive dysfunction after colorectal surgery. BMC Anesthesiol 2019;19:6. 30621601 10.1186/s12871-018-0676-4 PMC6325738

[132] Baek W , Kim YM , Lee H . Risk factors of postoperative delirium in older adult spine surgery patients: A meta‐analysis. AORN J. 2020;112:650‐661. 33252809 10.1002/aorn.13252

[133] Yang Y , Zhao X , Dong T , Yang Z , Zhang Q , Zhang Y . Risk factors for postoperative delirium following hip fracture repair in elderly patients: A systematic review and meta‐analysis. Aging Clin Exp Res. 2017;29:115‐126. 10.1007/s40520-016-0541-6 26873816

[134] Guan H‐L , Liu H , Hu X‐Y , et al. Urinary albumin creatinine ratio associated with postoperative delirium in elderly patients undergoing elective non‐cardiac surgery: A prospective observational study. CNS Neurosci Ther;n/a. 10.1111/cns.13717 PMC8928921 34415671

[135] Zhang J , Bi J‐J , Guo G‐J , et al. Abnormal composition of gut microbiota contributes to delirium‐like behaviors after abdominal surgery in mice. CNS Neurosci Ther. 2019;25:685‐696. 30680947 10.1111/cns.13103 PMC6515708

[136] Sun Y , Feng H , Zou T , et al. Assessment of risk factors for postoperative cognitive dysfunction after coronary artery bypass surgery: A single‐center retrospective cohort study. Biosci Rep 2021;41:BSR20190719. 33511391 10.1042/BSR20190719 PMC7901012

[137] Grocott HP , Mackensen GB , Grigore AM , et al. Postoperative hyperthermia is associated with cognitive dysfunction after coronary artery bypass graft surgery. Stroke. 2002;33:537‐541. 11823666 10.1161/hs0202.102600

[138] Xiao H , Run X , Cao X , et al. Temperature control can abolish anesthesia‐induced tau hyperphosphorylation and partly reverse anesthesia‐induced cognitive impairment in old mice. Psychiatry Clin Neurosci. 2013;67:493‐500. 23992430 10.1111/pcn.12091

[139] Uysal Aİ , Altıparmak B , Yaşar E , et al. The effects of early femoral nerve block intervention on preoperative pain management and incidence of postoperative delirium geriatric patients undergoing trochanteric femur fracture surgery: A randomized controlled trial. Ulus Travma Acil Cerrahi Derg. 2020;26:109‐114. 31942744 10.14744/tjtes.2019.78002

[140] Kanno M , Doi M , Kubota K , Kanoya Y . Risk factors for postoperative delirium and subsyndromal delirium in older patients in the surgical ward: A prospective observational study. PLoS One. 2021;16:e0255607. 34339463 10.1371/journal.pone.0255607 PMC8328296

[141] Bramley P , McArthur K , Blayney A , McCullagh I . Risk factors for postoperative delirium: An umbrella review of systematic reviews. Int J Surg. 2021;93:106063. 34411752 10.1016/j.ijsu.2021.106063

[142] Wei W , Chen X , Yu J , Li XQ . Risk factors for postoperative stroke in adults patients with moyamoya disease: a systematic review with meta‐analysis. BMC Neurol. 2019;19:98. 31092214 10.1186/s12883-019-1327-1 PMC6518622

[143] Wahlsten LR , Zareini B , Smedegaard L , Gislason GH , Palm H , Brorson S . A medical history of arterial thrombosis is a strong predictor of post‐operative myocardial infarction and stroke in patients with hip fractures—a nationwide cohort study. Age Ageing. 2021;50:1252‐1260. 33507243 10.1093/ageing/afaa279

[144] Li L‐Z , Huang Y‐Y , Yang Z‐H , Zhang S‐J , Han Z‐P , Luo Y‐M . Potential microglia‐based interventions for stroke. CNS Neurosci Ther. 2020;26:288‐296. 32064759 10.1111/cns.13291 PMC7052807

[145] Okita Y , Ikeno Y , Yokawa K , et al. Direct perfusion of the carotid artery in patients with brain malperfusion secondary to acute aortic dissection. Gen Thorac Cardiovasc Surg. 2019;67:161‐167. 29285704 10.1007/s11748-017-0873-y

[146] Zhao H , Guo F , Xu J , et al. Preoperative imaging risk findings for postoperative new stroke in patients with acute Type A aortic dissection. Frontiers in Cardiovascular Medicine 2020;7. 10.3389/fcvm.2020.602610 PMC7734126 33330666

[147] Zhao Q , Yan T , Chopp M , Venkat P , Chen J . Brain‐kidney interaction: Renal dysfunction following ischemic stroke. J Cereb Blood Flow Metab. 2020;40:246‐262. 31766979 10.1177/0271678X19890931 PMC7370616

[148] Ghoshal S , Gomez J , Datar SV , Tegeler C , Sarwal A , Freedman BI . The impact of chronic kidney disease on cerebral hemodynamics: A transcranial Doppler study. J Cereb Blood Flow Metab. 2020;40:482‐487. 31842666 10.1177/0271678X19893337 PMC7026848

[149] Arsenault KA , Yusuf AM , Crystal E , et al. Interventions for preventing post‐operative atrial fibrillation in patients undergoing heart surgery. Cochrane Database Syst Rev. 2013. 10.1002/14651858.CD003611.pub3 PMC7387225 23440790

[150] Wang KKP , Liu W , Chew STH , Ti LK , Shen L . New‐onset atrial fibrillation after cardiac surgery is a significant risk factor for long‐term stroke: an eight‐year prospective cohort study. J Cardiothorac Vasc Anesth. 2021;35(12):3559‐3564. 34330576 10.1053/j.jvca.2021.07.003

[151] Junejo RT , Braz ID , Lucas SJ , et al. Neurovascular coupling and cerebral autoregulation in atrial fibrillation. J Cereb Blood Flow Metab. 2020;40:1647‐1657. 31426699 10.1177/0271678X19870770 PMC7370373

